# Classification of human cancers based on DNA copy number amplification modeling

**DOI:** 10.1186/1755-8794-1-15

**Published:** 2008-05-14

**Authors:** Samuel Myllykangas, Jarkko Tikka, Tom Böhling, Sakari Knuutila, Jaakko Hollmén

**Affiliations:** 1 Department of Pathology, Haartman Institute and HUSLAB, University of Helsinki and Helsinki University Central Hospital, P.O. Box 21, FI-00014, University of Helsinki, Helsinki, Finland; 2 Department of Information and Computer Science, Helsinki University of Technology, P.O. Box 5400, FI-02015 TKK, Espoo, Finland

## Abstract

**Background:**

DNA amplifications alter gene dosage in cancer genomes by multiplying the gene copy number. Amplifications are quintessential in a considerable number of advanced cancers of various anatomical locations. The aims of this study were to classify human cancers based on their amplification patterns, explore the biological and clinical fundamentals behind their amplification-pattern based classification, and understand the characteristics in human genomic architecture that associate with amplification mechanisms.

**Methods:**

We applied a machine learning approach to model DNA copy number amplifications using a data set of binary amplification records at chromosome sub-band resolution from 4400 cases that represent 82 cancer types. Amplification data was fused with background data: clinical, histological and biological classifications, and cytogenetic annotations. Statistical hypothesis testing was used to mine associations between the data sets.

**Results:**

Probabilistic clustering of each chromosome identified 111 amplification models and divided the cancer cases into clusters. The distribution of classification terms in the amplification-model based clustering of cancer cases revealed cancer classes that were associated with specific DNA copy number amplification models. Amplification patterns – finite or bounded descriptions of the ranges of the amplifications in the chromosome – were extracted from the clustered data and expressed according to the original cytogenetic nomenclature. This was achieved by maximal frequent itemset mining using the cluster-specific data sets. The boundaries of amplification patterns were shown to be enriched with fragile sites, telomeres, centromeres, and light chromosome bands.

**Conclusions:**

Our results demonstrate that amplifications are non-random chromosomal changes and specifically selected in tumor tissue microenvironment. Furthermore, statistical evidence showed that specific chromosomal features co-localize with amplification breakpoints and link them in the amplification process.

## Background

Alterations that increase DNA copy number are frequently observed in a variety of human cancers [1,2]. An amplification is a mutation that increases the copy number of a specific DNA segment in a cancer cell [3,4]. A normal diploid genome contains two DNA copies, while amplification increases the DNA copy number [5]. High-level gene amplifications may significantly elevate the gene copy number, e.g., the amplifications of *MYC *and *EGFR *oncogenes have been shown to be more than hundred-fold in neuroblastoma [6] and gliomas [7]. Gene amplifications have clinical relevance as targets for therapy and in predictive diagnosis. For example, amplification of the *ERBB2 *gene is an indicator for trastuzumab (Herceptin^®^) treatment of patients with metastatic breast cancer. In general, cancers with DNA copy number amplifications have worse prognosis and poorer survival than cancers that do not manifest amplifications. The amplifications have been shown to associate with adverse clinical outcomes, i.e., high grade and advanced stage, metastasis, and poor response to therapy [8].

The guidelines for classification of tumors have been established by the World Health Organization (WHO) [9]. The WHO classification is based on the evaluation of the primary organ site, morphology, cell type, histology, and malignancy state. In addition, the epidemiological, etiological, clinical, and genetic features of tumors have been evaluated. In a subset of hematologic malignancies, specific mutations and translocations have been used in classification but otherwise tumor classification is based on clinical, histological, and pathological parameters. Although a wide range of cancer subtype-specific genetic abnormalities are known, they are rarely used to classify cancers. Given that genomic changes underlie the cancer phenotype, DNA copy number amplification is a justified foundation for classification. Nonetheless, molecular properties underlie phenotypic changes in cancer cells and contribute to the clinical outcome. Thus, molecular classification of cancers is well-founded. DNA copy number amplifications are suitable classification targets, because they are relatively prevalent in a variety of cancers. Since 1992, it has been possible to screen DNA copy number amplifications in genome-wide coverage using comparative genomic hybridization (CGH) [10] and large amounts of DNA copy number data from different cancers have been published and collected [1,11].

In a previous amplification profiling study, we identified four separate clusters and showed that the clusters based on DNA copy number amplifications comprised anatomically similar neoplasms [1]. For example, gastrointestinal adenocarcinomas (gastric cancer, colorectal cancer, and Barrett's adenocarcinoma) clustered together. Similar clustering emerged when amplification-activated oncogenes were analyzed using hierarchical clustering [8]: cancers with similar embryonic background (hematopoietic, mesenchymal or epithelial) formed separate clusters. Even though cancer types inside the identified clusters showed similar biological backgrounds, using the profiling approach, specific amplifications could not be appointed for specific cancer classes and analytical assessment was not plausible. Here, we used probabilistic modeling and a collection of DNA copy number amplifications, and identified 111 specific amplification models. Modeling was performed based on mixtures of multivariate Bernoulli distributions (see sub-section of methods entitled "Probabilistic modeling of DNA copy number amplification" for details). Based on the amplification modeling, the cancer cases were divided into clusters. Specific cancer cases, either of the same type or etiology, were shown to associate with specific amplification model-based clusters. Compact and comprehensible presentations for probabilistic amplification models, amplification patterns, were extracted using maximal frequent itemset mining (see sub-section of methods entitled "Finite descriptions for continuous DNA copy number amplification models" for details). Amplification patterns represent the ranges and structures of the amplicons. We present statistical evidence showing that fragile sites, telomeres, centromeres, and light chromosome bands are enriched at the amplicon boundaries, linking them to the mechanisms of amplification.

## Methods

### DNA copy number amplification and cancer classification data

DNA copy number amplification data were retrieved from [12]. The compilation of DNA copy number amplification data contains curated data from more than 800 published CGH studies on 4590 cases [1]. The data set includes DNA copy number amplification data at chromosome sub-band resolution (393 bands). The original classification of 73 human neoplasms was redefined to contain 95 specific neoplasm types by sub-classifying B-cell neoplasms and neuroepithelial tumors. The studied neoplasms were grouped according to the guidelines provided in the WHO Classification of Tumors [9]. In addition to the WHO classification, the neoplasms were arranged according to cell-lineage, which included determination of system, organ, cell type, and embryonic lineages. Moreover, the neoplasm types were categorized using clinical and genetic attributes. Gender and age group specificity was determined based on the WHO classification. Various etiological factors were collected from the WHO classification: tobacco, alcohol, hormonal imbalance, ultraviolet radiation, obesity, diet, human immunodeficiency virus, AIDS, human papilloma virus, Epstein-Barr virus, polyoma virus, bacterial and parasite infections, radiation, and prosthetic implant, as well as asbestos exposure and toxin exposures. Inflammatory etiologies of neoplasms and underlying conditions were defined according to literature. Tumor behavior was defined according to the stage (cancer, benign, and border line). After the neoplasms were filtered to include only malignant cancers and to discard benign, borderline, and non-malignant tumors, the number of cancer types was 82.

### Probabilistic modeling of DNA copy number amplification

We applied machine learning techniques to model DNA copy number amplifications. The goal of probabilistic modeling is to estimate an unknown probability distribution based on observations to describe the inherent structure in the data. Finite mixture models are powerful and widely used in the estimation of complex probability distributions [13,14]. The advantage of a mixture model is that its components can represent different parts of the true distribution, which would be impossible to estimate by a single parametric distribution. In this work, we concentrated on the mixtures of multivariate Bernoulli distributions, since the representation of our DNA copy number amplification data was binary.

Analysis of DNA copy number amplification data was carried out separately for each human chromosome (except chromosome Y). Due to the low number of observations and insufficient resolution, the Y chromosome was excluded from the analysis. The mixture models were constructed separately for each chromosome, since only 10 percent of the observations showed amplification in more than one chromosome. The observations with amplification in several chromosomes were included in the analysis of corresponding chromosomes. In brief, DNA copy number amplification data of cancers including *N *= 4402 cases were used in the modeling. DNA copy number amplification data can be presented as binary vectors *x *∈ {0,1}^*d *^in which *x*_*i *_= 1 denotes an amplified chromosome band and *x*_*i *_= 0 stands for a non-amplified band and *d *is the number of bands. The probabilities of the outcomes of observation ***x ***= (*x*_1_,..., *x*_*d*_) were modelled as *θ*_*i *_= *P*(*x*_*i *_= 1),*i *= 1,..., *d*. Probability of the observed vector ***x ***was estimated using the finite mixture of multivariate Bernoulli distributions

$p\left(x|\Theta \right)=\sum_{j=1}^{J}\pi_{j}p\left(x|\theta_{j}\right)=\sum_{j=1}^{J}\pi_{j}\prod_{i=1}^{d}\theta_{ji}^{x_{i}}{\left(1-\theta_{ji}\right)}^{1-x_{i}}$, where $\Theta =\left\{J,{\left\{\pi_{j},\theta_{j}\right\}}_{j=1}^{J}\right\}$ denotes the parameters of the model. The multivariate Bernoulli distributions *p*(***x***|***θ ***_*j*_), *j *= 1,..., *J*, also called the component distributions, are parameterized by ***θ***_*j *_= (*θ*_*j*1_,..., *θ*_*jd*_) and *π*_*j *_are mixture proportions with the properties *π*_*j *_≥ 0 and $\sum_{j=1}^{J}\pi_{j}=1$. Although the finite mixture of multivariate Bernoulli distributions have been shown to be non-identifiable [15], they are useful in practical estimation problems [16].

In the case of *N *observations ***x***^*n*^, *n*= 1,..., *N *and *J *mixture components, the maximum likelihood estimates of the parameters ${\left\{{\hat{\pi }}_{j},{\hat{\theta }}_{j}\right\}}_{j=1}^{J}$ are obtained by maximizing the log-likelihood of the observations $l=\sum_{n=1}^{N}\log\left[\sum_{j=1}^{J}\pi_{j}\prod_{i=1}^{d}\theta_{ji}^{x_{ni}}{\left(1-\theta_{ji}\right)}^{1-x_{ni}}\right]$. The optimization was carried out using the Expectation-Maximization (EM) algorithm [17-19]. The derivation of the EM algorithm for the finite mixture of multivariate Bernoulli distributions has been explained in detail by Everitt and Hand [14]. In the E-step, the posterior probabilities that the *j*th component distribution has generated the data point ***x***_*n *_are evaluated. In the M-step, the values of parameters ${\left\{{\hat{\pi }}_{j},{\hat{\theta }}_{j}\right\}}_{j=1}^{J}$ are updated using the evaluated posterior probabilities. The iteration between E- and M-steps gives monotonically increasing series of the values for the log-likelihood. The EM algorithm was terminated when the relative change in log-likelihood was smaller than10^-4^. According to Carreira-Perpiran and Renals, and Tikka et al. [16,20], the initial values of parameters *θ*_*ji*_, *j *= 1,..., *J*, *i *= 1,..., *d *were selected randomly from range 0.25–0.75 and the initial values of mixture proportions were *π*_*j *_= 1/*J*.

In order to select a model with an appropriate complexity, the number of component distributions *J *was selected using 5-fold cross validation [21] that was repeated 10 times varying *J *from 2 to 30. The selected number of components maximized the validation log-likelihood, except in five cases, when less complex models were chosen to achieve validation log-likelihood that was in practice as good as the estimated optimum. Less complex models were chosen for chromosomes 3, 7, 8, 17, and 21. The final mixture model, with the number of component distributions based on cross validation, was trained 5 times and the model maximizing the log-likelihood was selected. The repetitions were done to avoid the local maxima in the log-likelihood. The obtained component distributions were regarded as DNA copy number amplification models.

### Data mining from DNA copy number amplification model-based clusters

We applied data mining techniques and WHO derived cancer classifications as background data to explore the amplification model-based clustering of cancer cases. Cancer cases were divided into separate clusters for each chromosome using the inherent structure of DNA copy number amplification models. The component distributions of mixture model define clusters. Following the probabilistic approach, each observation was allocated to cluster j*, which maximizes the posterior probability according to Bayes's theorem $j^{*}=\underset{j}{\arg\max}\frac{p\left(j\right)p\left(x|j\right)}{p\left(x\right)}=\underset{j}{\arg\max}\pi_{j}\prod_{i=1}^{d}\theta_{ji}^{x_{i}}{\left(1-\theta_{ji}\right)}^{1-x_{i}}$. Then, the observations belonging to cluster *j *were characterized by the corresponding component distribution ***θ***_*j*_. After clustering, the data in each cluster were divided into a test group and a reference group according to the collected classification terms. The test group contained those cases that were associated with a specific classification term (e.g., tobacco-related) and the reference group contained all other cases (not related to tobacco). Proportions of observations in each cluster *f*_*j*1 _and *f*_*j*0 _were calculated for the test and the reference group, respectively. The differences in cluster-specific observation proportions were compared by performing a pooled proportions statistical test [22]. The null hypothesis *H*_0_ stated that the proportions are equal *f*_*j*1 _= *f*_*j*0 _and alternative hypothesis *H*_1 _was that the proportion in the test group is larger than that in the reference group *f*_*j*1 _> *f*_*j*0 _or vice versa. The test statistic for the pooled proportions test is defined as $z=\frac{f_{j1}-f_{j0}}{\sqrt{\hat{f}\left(1-\hat{f}\right)\left(1/n_{1}+1/n_{0}\right)}}$, where $\hat{f}=\frac{f_{j1}n_{1}+f_{j0}n_{0}}{n_{1}+n_{0}}$ and *n*_1 _and *n*_0 _are the number of observations in the test group and in the reference group, respectively. The test statistic *z *is approximately normally distributed with zero mean and unit variance. The described test was carried out for each amplification pattern and classification term.

Due to the large number of hypotheses, we used the following procedure for control of the false discovery rate [23]. Let $p_{r_{1}}\leq p_{r_{2}}\leq ...\leq p_{r_{m}}$ denote the observed ordered unadjusted *p*-values, where *m *is the number of hypotheses (*m *= 13659). For control of the false discovery rate at level *α *search $i^{*}=\max\left\{i:p_{r_{i}}\leq \frac{i}{m}\alpha \right\}$.

The null hypotheses are rejected in the case of *i *≤ i*. In the experiments, we used the significance level *α *= 0.001, which corresponded to the unadjusted *p*-value 0.00005. Thus, the alternative hypothesis was accepted, i.e., the difference was regarded statistically significant, when the *p*-value of the test was lower than 0.00005.

### Finite descriptions for continuous DNA copy number amplification models

Fusing of amplification models with relevant genomic mapping data required that continuous models were transformed into compact representations in the original nomenclature of the chromosomal bands. Finite descriptions, namely amplification patterns, were formed using maximal frequent itemset mining as presented earlier [24]. For the definitions and notations used in the following brief technical description of maximal frequent itemsets, we refer to Burdick et al. [25]. In a binary database with ***x***_***i ***_= {0,1}^*d *^and index set *I *= {1,...*d*}, an itemset is a subset of the index set *I *and a *k*-itemset is an itemset with cardinality *k *[25]. Support for an itemset *X *is defined as the frequency of the rows in the database including the items in *X*, i.e., how often *X *occurs in the database. *σ *is a predefined parameter that sets a threshold for selecting frequent itemsets. The mining task is to find all itemsets that have a frequency higher than *σ*. These are regarded as frequent itemsets. If an itemset *X *is frequent and no superset of *X *is frequent, we say that *X *is a maximal frequent itemset. The implementation by Burdick et al. [25] integrates a depth-first traversal of the itemset lattice with effective pruning mechanisms that significantly improve mining performance. We use maximal frequent itemsets in summarizing the marginal distribution of the clusters in a compact and understandable manner. In mining for the amplification patterns in the clustered data sets, we used a frequency threshold of *σ *= 0.5, so that the amplification patterns would be representative of the clusters in question. In simple terms, amplification patterns portray amplification models using finite ranges that capture the chromosomal structure of the amplified DNA element (also referred to as amplicon). The resulting amplification patterns are collections of the largest sets of chromosomal bands that occur jointly (together) in more than half of the data cases. The amplification patterns are thought to be representative of the whole cluster. As such, they represent the most probable amplicon structures and were used to map amplicon boundaries and putative DNA double-strand breakpoints.

### Data mining from amplification patterns

A way to investigate the nature of DNA copy number amplification patterns is to compare them with relevant cytogenetic background data. For data mining, amplification patterns were used to depict the amplicon structures and identify putative ends of the amplified regions. Cytogenetic features on amplicon boundaries were characterized to elucidate the genomic features that predispose to DNA double-strand breaks and enable amplification. Mechanistic models of amplification predict that DNA double-strand breaks must occur at the ends of the amplified chromosomal element. Enrichment of specific chromosomal regions in the ends of the amplification patterns (putative amplicons) was tested. The tested chromosomal regions included fragile sites, telomeres, centromeres, light and dark G-bands, and variable regions. Fragile sites were mapped according to the National Center for Biotechnology Information database annotations. Chromosome bands mapping to telomeric, centromeric and variable regions, as well as dark and light bands were extracted from the International System for Human Cytogenetic Nomenclature (2005) annotations [26]. We were interested in knowing whether the ends of amplification patterns were involved in an unexpectedly large number of specific chromosomal features, which would provide a possible explanation for DNA breakage associated with amplification. The test statistics were the differences between frequencies of chromosomal features in different sites of a given amplification pattern (borders, inside, and in general). If many patterns are based on one component distribution of the Bernoulli mixture model, they are very likely to overlap. In cases like this, the hypothesis testing includes the band in both sets with equal importance. We executed a permutation test of 10000 iterations to the hypothesis of comparing the proportions of chromosomal features. When executing the permutation test, an equal number of random bands to the number of bands of the chromosomal feature that was tested were distributed along the amplification patterns. Then, the corresponding proportions of the randomized sites occurring in the border and inside bands were calculated. This can be seen as a means to obtain empirical samples of the test statistic under the null hypothesis. The difference in proportions of the true test statistic and the randomized reference could then be calculated. The *p*-value for the one-tailed test was calculated using the difference in the proportions of the test statistic and randomized reference values. The threshold for significant findings was *p *< 0.05.

## Results

### Probabilistic models of DNA copy number amplification

**Figure 1:**
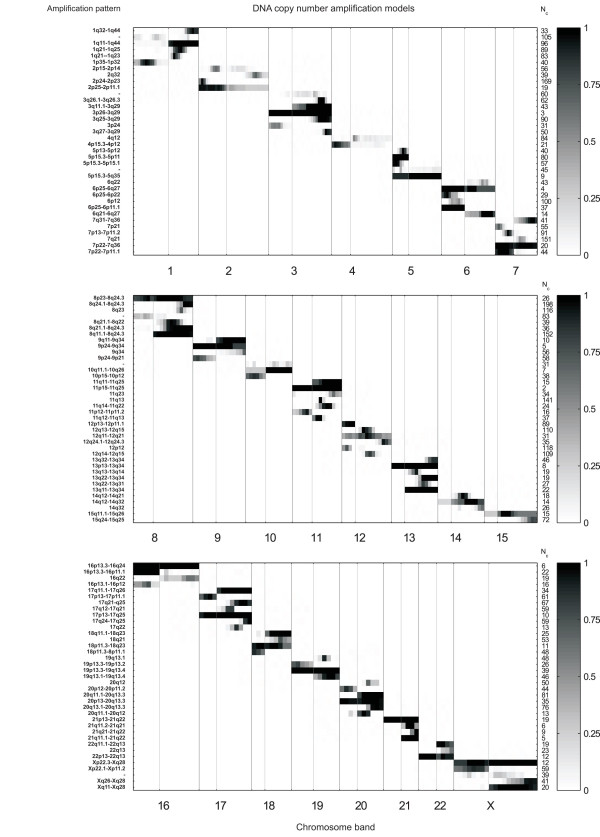
**Probabilistic models of DNA copy number amplification**. The models are component distributions of chromosome specific mixture models. Models are marked in the figure on separate lines. The probability of an amplification in each chromosome band is denoted using white to black scaling, where black indicates a chromosome band with a high probability of an amplification (*p *= 1) and white indicates a chromosome band with a low probability of an amplification (*p *= 0). Probabilities between 0 and 1 have been linearly scaled as shades of gray. Amplification patterns (based on the maximal frequent itemsets) are reported on the left side of the amplification models. Prevalence of the amplification patterns in terms of the number of cancer cases (N_c_) is shown on the right side of the models.

**Figure 2:**
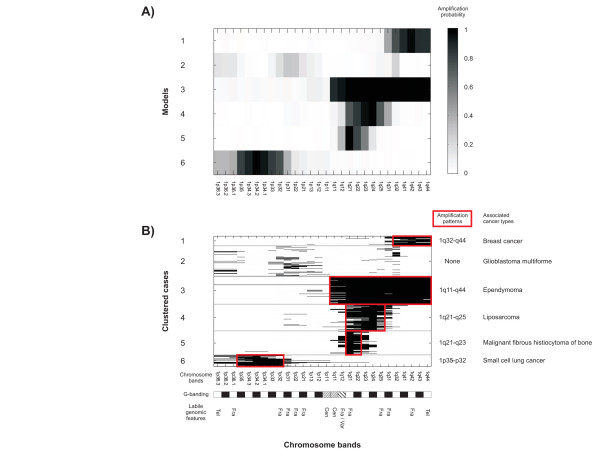
**Amplification models and clustered data from chromosome 1**. A) Component distributions of the amplification model and B) Clustered DNA copy number amplification data are shown for chromosome 1. Amplification models are presented as amplification probabilities in each chromosome band. White to black scaling represents amplification probabilities from zero to one. Six component distributions of the amplification model (panel A) have been used to cluster data into six clusters (panel B). In panel B, each line represents a single cancer case in the data set and amplified chromosome bands are marked black. Models and clusters are separated using gray horizontal lines. Amplification patterns are marked in the figure by red boxes and cancer types that are associated with the amplification model-based cancer clusters are denoted in the figure. Chromosome band annotations were collected from the International System for Human Cytogenetic Nomenclature (2005) [26] and are marked below corresponding chromosome bands. Black and white chromosome bands according to G-banding are marked. In addition, chromosome bands with specific chromosomal properties, namely, centromeres (Cen), telomeres (Tel), fragile sites (Fra), and variable regions (Var), are marked.

We identified 111 amplification models (Figure 1). The number of clusters in chromosomes 1–22 and X varied between 2 and 7: chr1 (6), chr2 (4), chr3 (7), chr4 (2), chr5 (5), chr6 (6), chr7 (6), chr8 (7), chr9 (4), chr10 (3), chr11 (7), chr12 (6), chr13 (6), chr14 (3), chr15 (2), chr16 (4), chr17 (7), chr18 (4), chr19 (4), chr20 (6), chr21 (4), chr22 (3), and chrX (5). The Y chromosome was omitted from the analysis due to the low number of cases. Figure 1 shows the number of cancer cases (N_c_) included in each amplification model. Vectors of probability parameters ***θ***_j _represent the resulting amplification models, where each mixture component assigns a continuous probability value *θ*_ji _to each chromosome band. One chromosome band may have non-zero probabilities in different components, since components may correspond to different amplicon structures. Figure 2 shows that the clustering based on the probabilistic model of DNA copy number amplification manages to predetermine the structure of amplifications. It represents a typical amplification pattern and the general properties observed in an amplification, which may encompass additional bystander areas around the target gene locus.

### DNA copy number amplification model-based clustering of cancer cases

The identified DNA copy number amplification models can be used to divide cases into clusters with similar molecular aberration. Figure 2 shows the data from chromosome 1 for reference. The clusters based on DNA copy number amplification models can be fused with known background data of the cancer types to study the underlying specificity of the amplifications.

**Figure 3:**
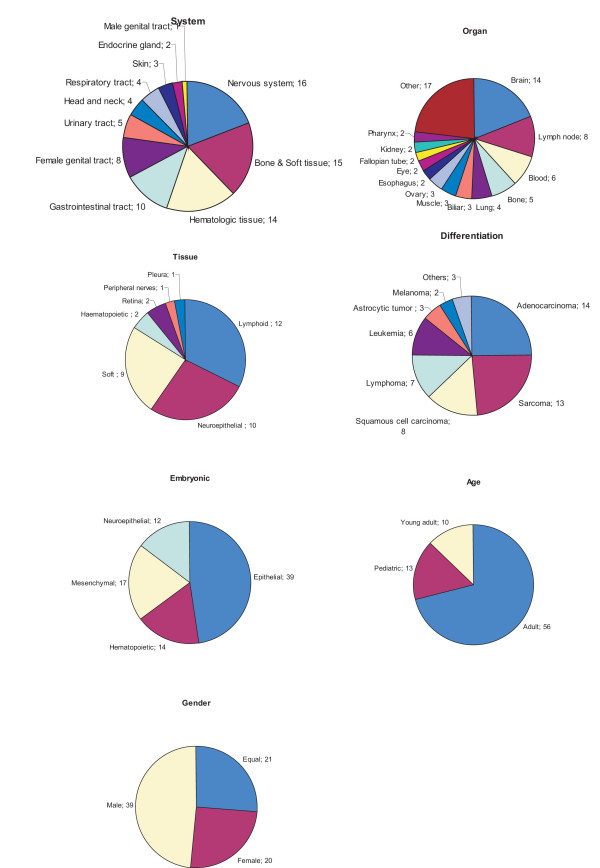
**Distribution of cancer classification attributes and classification terms**. Classification data was compiled from the WHO sources [9]. Figure is divided to individual pie charts according to different classification attributes. Each pie chart describes the numbers of cancer types in specific classification terms.

**Figure 4:**
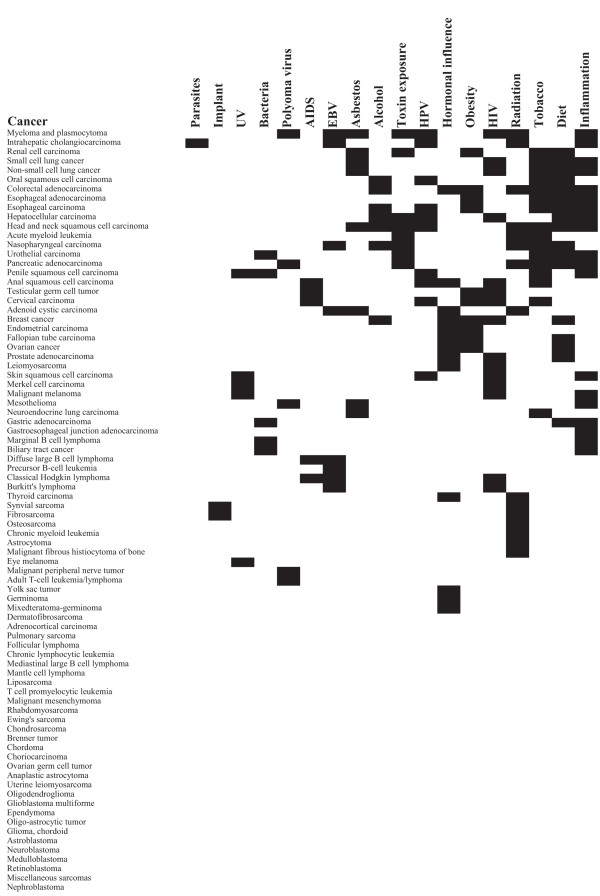
**Etiological factors of cancers**. Etiological data was compiled from the WHO sources [9]. Each row describes a cancer type and the etiological factors that have been associated with it (indicated by black boxes). Cancer type rows and etiological factor columns are sorted according to hierarchical clustering. Between groups-linkage method and Squared Euclidean distance measure for binary classification terms were used in clustering.

Classification data of 95 human neoplasms were collected from the literature [see Additional file 1]. The analysis was restricted to malignant cancers of 82 different cancer types. Figure 3 presents the classification distribution based on cell lineages, age, and gender. Classification based on cell lineage contained anatomical system, organ, tissue, differentiation, and embryonic lineages. These attributes were divided into classification terms, e.g., nervous system (anatomical system), brain (organ), and glioma (cell). The classification terms can partially overlap with different attributes. Differentiation lineage (e.g., adenocarcinoma) refers to the histological type of the malignancy. Embryonic lineage divides cases into four main developmental compartments: epithelial, mesenchymal, hematopoietic, and neuroepithelial. The clinical attributes were age (pediatric, young adults, and adults) and gender specifications. In addition, 19 different etiological factors were collected (Figure 4). In all, 29 attributes and 100 classification terms were accumulated. Classification terms were appointed for cancers as primary data of individual cases was not available in the amplification data compilation. The compilation of DNA copy number amplification data was revised regarding the new annotations [12].

**Figure 5:**
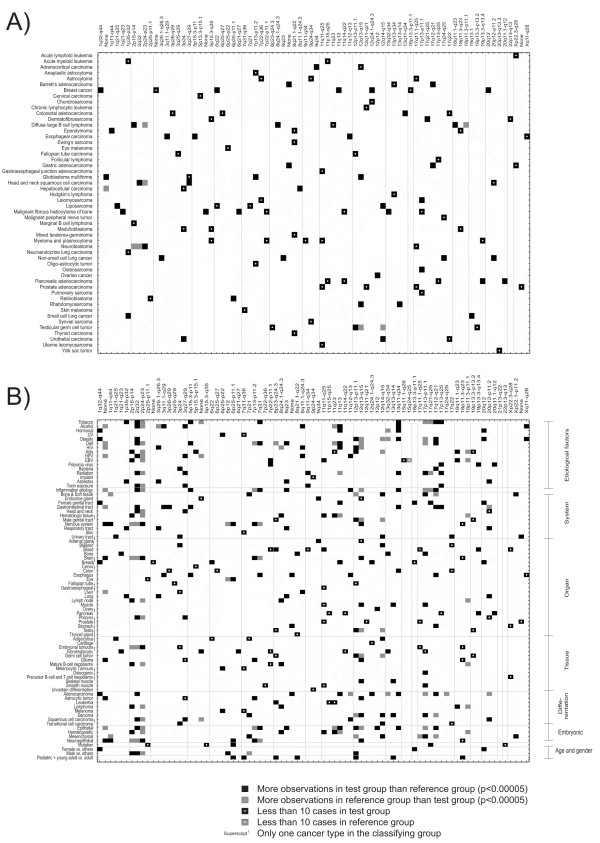
**Associations between DNA copy number amplification models and classification terms**. Data are presented for A) individual cancer types and B) classification terms. Data was collected from the WHO sources [9]. Rows show A) cancer types or B) specific classification terms. Each column represents one amplification pattern. Statistically significant differences in the prevalence of the test group (cancer cases or classification terms) and reference group (other cases) are marked with black and gray boxes. Findings that are based on a small sample (<10) are marked with a white circle inside the black box. Only amplification patterns, cancer types and classification terms with significant findings are shown.

Frequencies of classification annotation terms were compared between cases in the studied cluster and a reference group that contained all other cases. The statistical significance of difference in the frequencies observed in each amplification model based cluster was determined using a hypothesis test. The significance threshold was set to 0.00005 and *p*-values were corrected for multiple testing using the Benjamini-Hochberg false discovery rate method [23]. Statistically significant observations are presented in Figure 5. Individual cancers (Figure 5A) and sample groups with specific classification terms (Figure 5B) were tested against all other samples. Our results show that a subset of amplifications is associated with specific cancer type, whereas some amplifications are more commonly shared by cancers of similar etiology or cell-lineage. For example, 1q33-q44 amplification is specific to breast cancers and 17q12-q21 region is specifically amplified in gastric cancer and Barrett's adenocarcinoma (Figure 5A). Similarly, 1q32-q44 amplification is specifically present in cancers associated with hormonal imbalance, obesity, female genital tract, breast tissue, and adenocarcinoma as well as cancers with female overrepresentation (Figure 5B). On the other hand, 17q12-q21 amplicon is enriched in cancers that associate with tobacco, obesity, diet, bacterial infections, inflammation, gastrointestinal tract, esophagus, stomach, adenocarcinoma, epithelial origin, and ethnic prevalence (Figure 5B).

### Pattern description for probabilistic models of DNA copy number amplification

In order to facilitate the interpretation, we generated compact and understandable descriptions of the amplification models. The descriptions, i.e., amplification patterns, are local, finite, and plausible representations of the chromosomal areas of amplification. The amplification patterns were identified as maximal frequent itemsets and the chromosome bands are expressed following the original cytogenetic nomenclature. Amplification patterns depict the structures of the amplicons. One amplification model may result in many patterns. In fact, we extracted 140 maximal frequent itemsets from the 111 amplification models. When multiple patterns were identified for a specific amplification model, the most frequent pattern was chosen. Amplification patters are reported alongside their representative models in Figure 1. Amplification patterns in chromosome 1 are marked in Figure 2.

### Mechanisms of DNA copy number amplification

Amplification patterns were used to investigate the mechanisms of DNA copy number amplification. Our hypothesis was that patterns represent the general structures of amplicons and can thus be applied to map amplicon boundaries and genomic loci that are susceptible to DNA double-strand damage. Differences in the proportions of labile chromosomal features in amplification patterns and within border bands or inside amplification patterns were determined using a hypothesis test (Table 1). Noteworthy is that the same band can occur both inside and on the border of the patterns, which can overlap. This is exemplified in Figure 2, where the ends of one pattern are inside another pattern. To be precise, the telomeric ends of the amplification patterns for models four and five (1q23 and 1q25, respectively) are inside the amplification pattern of model three (1q11-1q44). Statistically significant differences in proportions (*p*-value < 0.05) were identified when the proportions of fragile sites within the borders of amplification patterns were compared with the proportions of fragile sites in the amplification patterns. Similarly, light chromosome bands, telomeres, and centromeres were more frequent in the border bands than in the patterns in general. Dark chromosome bands inside the amplification patterns were more frequent than those in patterns as a whole.

**Table 1:** Hypothesis testing of proportions of labile chromosomal sites within the amplification patterns.

**Labile chromosomal site**	**Proportion in patterns**	**Proportion in amplification pattern borders**	**Proportion inside amplification patterns**	***p*-value**
Fragile sites	0.3086	0.3693	-	0.0069*
	0.3086	-	0.2975	0.8448
Dark chromosome bands	0.3739	0.3182	-	0.9896
	0.3739	-	0.4301	0.0000*
Light chromosome bands	0.4629	0.5114	-	0.0405*
	0.4629	-	0.4122	1.0000
Telomere bands	0.1217	0.2330	-	0.0000
	0.1217	-	0.0000	1.0000**
Centromere bands	0.1187	0.1477	-	0.0378*
	0.1187	-	0.1147	0.7481
Variable bands	0.0445	0.0227	-	0.9388
	0.0445	-	0.0430	0.7154

The proportion of chromosomal attributes in patterns in general is compared to the proportion of chromosomal attributes on the borders of the amplification patterns as well as inside amplification patterns (excluding the borders). Statistical significance of the difference in proportions was determined using permutation tests and *p*-values are marked in the table. Significant findings are marked with an asterisk.

*Statistically significant findings (*p *< 0.05).

**By definition, telomeres can not be located inside the amplification patterns and therefore this test could not yield any significant findings.

## Discussion

A machine learning approach was utilized to model DNA copy number amplifications in a landscape of cancers. The current modeling approach disregarded the cancer type information and modeled amplifications based on case-specific data vectors. This resulted in identification of 111 amplification models (Figure 1). The identified models could be viewed as specific cancer classes that allow more refined dissection of amplification processes. Our hypothesis was that cancers with a common amplification model might exhibit dependency on a specific oncogene amplification and therefore share common biological background. We tested this hypothesis by analyzing the distribution of WHO classification terms in the amplification modeling-based clustering of cancer cases. Specific amplification models were shown to associate with specific cancer types and classification terms (Figure 5). The non-random structure in the spectrum of DNA copy number amplifications in cancer suggests that cancer etiology and tumor microenvironment could manifest as specific amplification signatures. According to our results, amplifications are selected according to the anatomical locations and biological background of the cancers. Theoretically, carcinogenesis could be viewed as an evolutionary process that involves the selection of cancer cells in the somatic tissue by specific mutations. In the Darwinian perspective, the classification based on DNA copy number amplifications reflects the differences in the selective properties in different anatomical locations and in specific adaptation of cancers with similar biological backgrounds.

DNA copies generated in amplification manifest as concatenated homogenously staining regions and extra-chromosomal acentric DNA fragments, double minutes and episomes [3,27]. Models of DNA amplification mechanisms, the breakage-fusion-bridge cycle and excision of extrachromosomal DNA segments, state that two independent DNA double-strand breaks that flank the amplified region are required to initiate the amplification pathway [28]. Using the amplification modeling and pattern discovery approach, presented in the current study, fixing of finite amplicon structures became feasible and amplification patterns, representations of amplicon structures, were determined from the DNA copy number amplification models. The amplification patterns could then be used to identify specific chromosomal sites that associate with amplicon boundaries. By fusing the boundaries of amplification patterns with cytogenetic annotations of the genome it was possible to elucidate the features in the chromosomal structure and genomic architecture that predispose the genome to amplifications. The hypothesis was that specific genomic regions may be damage-prone and susceptible to DNA double-strand breaks. The statistical hypothesis testing demonstrated that fragile sites, light chromosome bands, telomeres, and centromeres were enriched in the ends of the amplicons (Table 1). This suggests that these sites might be associated with the amplification mechanisms and DNA double-strand breakage at amplicon boundaries. Fragile sites are damage-prone genomic regions when cells are treated with chemicals that interfere with replication [29], which makes it likely that they are often found at amplification breakpoints. In addition to fragile sites, chromosome ends are unstable and may produce double-strand DNA breaks due to telomere shortening during replication and cell division [30]. Similarly, centromere regions have been shown to be unstable and damage-prone upon replication stress [31], which might explain the accumulation of amplicon boundaries on them. Light chromosome bands were also enriched at amplicon boundaries. The light bands contain euchromatin and are gene-rich, G/C-rich and late-replicating, whereas dark bands correspond to gene-poor, A/T-rich and early replicating heterochromatin. Due to its high gene content, the structure of euchromatin is more open than that of heterochromatin [32], which may affect its physical protection and render euchromatin more susceptible to DNA damage than the gene-poor heterochromatin. We hypothesize that open chromatin reduces the protection of chromosomal DNA and serves as preferential target for DNA damage. Open chromatin might therefore expose light chromosome bands to DNA double-strand breaks that initiate the amplification pathways.

## Conclusions

We classified human cancers based on DNA copy number amplification models. Cancer cases were fused with the WHO classification annotations. The inherent structure in the probabilistic clustering suggests that amplifications are non-randomly selected according to biological backgrounds of cancers. Amplification patterns were extracted and probed using cytogenetic annotations. We show statistical evidence that connects fragile sites, telomeres, centromeres, and light chromosome bands to the amplification mechanism. These results suggest that labile chromosomal features are involved in the amplification process by promoting the formation of DNA double-strand breaks at amplicon margins.

## Abbreviations

CGH: comparative genomic hybridization; WHO: World Health Organization; EM: Expectation-Maximization.

## Competing interests

The authors declare that they have no competing interests.

## Authors' contributions

SM, SK and JH conceived and designed the research and set goals. SM and TB collected clinical and biological background data. SM, JT and JH decided on the methodologies and planned data analysis. JT and JH performed computational work. SM, TB and SK interpreted the results. All authors participated in drafting of the manuscript and read and approved the final version. SM coordinated the research.

## Pre-publication history

The pre-publication history for this paper can be accessed here:



## Supplementary Material

Additional file 1 **Classification annotations for cancer types**. Supplement 1_Classification data.txt is a tab-delimited text-file containing all collected cancer classification data. Click here for file
